# Field evaluation of a rapid diagnostic test (Parascreen™) for malaria diagnosis in the Peruvian Amazon

**DOI:** 10.1186/1475-2875-9-154

**Published:** 2010-06-07

**Authors:** Jorge Bendezu, Angel Rosas, Tanilu Grande, Hugo Rodriguez, Alejandro Llanos-Cuentas, Jorge Escobedo, Dionicia Gamboa

**Affiliations:** 1 Instituto de Medicina Tropical "Alexander Von Humboldt", Universidad Peruana Cayetano Heredia, AP 4314, Lima 100, Peru; 2 Multi-Country Malaria Project "Malaria control on the cross border areas of the Andean Region: A community based approach"-PAMAFRO, Organismo Andino de Salud - Convenio Hipolito Unanue, Av. Paseo de la República 3832, Lima 27, Peru; 3 Direccion Regional de Salud de Loreto, Ministerio de Salud del Peru, Avenida 28 De Julio, Punchana - Iquitos — Loreto, Peru; 4 Departamento de Bioquímica, Biología Molecular y Farmacología, Facultad de Ciencias y Filosofía, Universidad Peruana Cayetano Heredia, AP 4314, Lima 100, Peru

## Abstract

**Background:**

The rapid diagnostic tests for malaria (RDT) constitute a fast and opportune alternative for non-complicated malaria diagnosis in areas where microscopy is not available. The objective of this study was to validate a RDT named Parascreen™ under field conditions in Iquitos, department of Loreto, Peru. Parascreen™ is a RDT that detects the histidine-rich protein 2 (HRP2) antigen from *Plasmodium falciparum *and lactate deshydrogenase from all *Plasmodium *species.

**Methods:**

Parascreen™ was compared with microscopy performed by experts (EM) and polymerase chain reaction (PCR) using the following indicators: sensitivity (Se), specificity (Sp), positive (PV+) and negative predictive values (PV-), positive (LR+) and negative likehood ratio (LR-).

**Results:**

332 patients with suspected non-complicated malaria who attended to the MOH health centres were enrolled between October and December 2006. For *P. falciparum *malaria, Parascreen™ in comparison with EM, had Se: 53.5%, Sp: 98.7%, PV+: 66.7%, PV-: 97.8%, LR+: 42.27 and LR-: 0.47; and for non-*P. falciparum *malaria, Se: 77.1%, Sp: 97.6%, PV+: 91.4%, PV-: 92.7%, LR+: 32.0 and LR-: 0.22. The comparison of Parascreen™ with PCR showed, for *P. falciparum *malaria, Se: 81.8%, Sp: 99.1%, PV+: 75%, PV-: 99.4, LR+: 87.27 and LR-: 0.18; and for non-*P. falciparum *malaria Se: 76.1%, Sp: 99.2%, PV+: 97.1%, PV-: 92.0%, LR+: 92.51 and LR-: 0.24.

**Conclusions:**

The study results indicate that Parascreen™ is not a valid and acceptable test for malaria diagnosis under the field conditions found in the Peruvian Amazon. The relative proportion of *Plasmodium *species, in addition to the genetic characteristics of the parasites in the area, must be considered before applying any RDT, especially after the finding of *P. falciparum *malaria parasites lacking *pfhrp2 *gene in this region.

## Background

Malaria affects people in more than 108 countries around the world, with nearly 243 million estimated cases and nearly 863 thousands of deaths reported in the last year [1]. Despite a reduction of the incidence by up to 40% during the last four years in Peru, malaria due to *Plasmodium falciparum *and *Plasmodium vivax *remains an important public health problem, especially in the Amazon region where more than 70% of the cases of the country are reported [2].

In malaria patients, a prompt and accurate diagnosis is the key for effective disease management, in order to reduce the probability of complications and mortality. The two diagnostic approaches currently in use, clinical diagnosis and microscopy, do not allow a satisfactory diagnosis of malaria. Clinical diagnosis, the most widely used, is unreliable because the symptoms of malaria are non-specific [3,4]. Diagnosis by microscopy, the established method for laboratory confirmation of malaria, presents technical and personnel requirements that often cannot be achieved [4-8], particularly in many areas of the Amazon region, far away from the main cities, where the population is widely dispersed and few health centres exist. Because of the reasons mentioned above and with the appearance of severe vivax malaria cases in different countries around the world in the last years [9], it is imperative to have a rapid and appropriate diagnosis method for malaria in Peru.

Rapid diagnostic tests for malaria (RDT) offer a good alternative with the advantage that it is an easy and rapid method, which requires minimum training [6,7,10-12]. The evaluation of different RDTs in many places in the last decade has demonstrated high sensitivity and specificity, compared with expert microscopy diagnosis as gold standard [3,6,8,10,11,13-15]. However, it was also observed that the same RDT used in different places showed different results, which is probably related to different conditions found in these places (temperature, humidity, characteristics of the malaria parasites, etc.). This is one of the reasons why initiatives like the WHO/TDR/FIND malaria RDT product testing programme, evaluating different RDTs under standardized conditions; could guide the malaria programmes in different countries to select the best RDT for a specific region [16].

Furthermore, the recent finding of *P. falciparum *field isolates lacking expression of the *pfhrp2 *gene in the Peruvian Amazon region should also be taken into account to choose the proper RDT for this region [17].

For more sensitive malaria detection, several polymerase chain reaction (PCR) assays have also been developed, and are mainly used in epidemiological studies [18]. The major advantage of this approach is the capability to detect malaria parasites in patients with low levels of parasitaemia, (five or less parasites per μL of blood), including sub-patent malaria infections, which can be detected with 100% of sensitivity and specificity. However, the difficulty in acquiring and maintaining the required technical skills is the main disadvantage of this technique [7,8,14].

Parascreen™ is a RDT that has been assessed in different studies in Asian and African countries [19-21], where the test performed well under field conditions. In the present study Parascreen™ was evaluated in different communities around Iquitos, department of Loreto, Peru, and compared its performance with expert microscopy and PCR.

## Methods

### Study design and specimens

Patients with history of fever with or without chills, sweating and headache (clinical symptoms suspicious of malaria), and with no history of anti-malarial treatment during the last two weeks, were enrolled through a passive malaria case detection in six health facilities around Iquitos (Department of Loreto) in the Peruvian Amazon, between October and December 2006. The minimum required sample size for this study was determined to be 96 confirmed malaria cases and 96 non-malaria cases, assuming a sensitivity and specificity at 90% with a precision of 6%, at a 95% confidence interval.

Blood samples for thick and thin blood films, for the Parascreen™ test and for PCR (collected on filter paper, 3 MM), were collected by finger-prick. Diagnosis procedures, including microscopy, Parascreen™ and PCR, were carried out by different staff blinded to each other result.

### Expert microscopy

This procedure was carried out in the six health centres, located in rural areas, using standard protocols according to the Peruvian national guidelines [22]. Thick smears prepared with a finger-prick sample were stained with 10% Giemsa and examined using a microscope with a 100 × oil immersion objective. The parasite density was expressed as the number of parasites/μl of blood, by using an average number of leucocytes per microlitre of blood of 6,000 (according to the Peruvian national guidelines) for the calculations (number of counted parasites, multiplied by 6,000, divided by the number of counted leucocytes, by counting a total of ≥200 and ≥500 leucocytes if the number of parasites per microscope field is > 10 and < 10, respectively) [22]. The results were recorded together with clinical and epidemiological data from each patient. For the quality control, 10% of the slides were examined by a second expert microscopist at the reference laboratory (Centro de Salud San Juan) in Iquitos.

### Parascreen™

Parascreen™(Zephyr Biomedical Systems, lot: 101051) is a RDT that detects the histidine-rich protein 2 antigen of *P. falciparum *and the lactate deshydrogenase of *Plasmodium*. The reactive strip has three different detection lines: a distal line with non-specific antibodies that recognize HRP-2 *P. falciparum*; a middle line with specific antibodies that recognize the lactate deshydrogenase enzyme of the "pan malaria group" (*P. falciparum *and non *-P. falciparum*), and a proximal line with antibodies that capture the excess of conjugate and therefore function as an assay control.

The RDT was performed and results were interpreted according to the manufacturer's instructions [23]. Briefly, only one pink-purple line in the proximal area (control line) means negative for malaria; one pink-purple line in the middle area, in addition to the control line, means non-*P. falciparum *infection; one pink-purple line, in addition to the previous two bands, means *P. falciparum *infection. When the control line did not appear or the interpretation was doubtful, the test was repeated.

### PCR procedures

The parasite DNA was extracted from the filter paper using the Chelex - 100 method (Bio-Rad Laboratories, Hercules, CA). Filter paper blood spots prepared with approximately 20 μL of blood were cut into pieces of approximately 5 mm^2^, and incubated with 20 μL of 0.05% saponin (Sigma™) at room temperature for four hours. Then 10 μL of 20% Chelex - 100 solution was added, and the sample was incubated for 10 minutes at 95°C, followed by a centrifugation at 11,000 g. The supernatant (DNA) was transferred into a new tube and stored at -20°C until use. The DNA was amplified by a semi-nested multiplex PCR method, as described by Rubio *et al *[24], The PCR products were analyzed in a 2% agarose gel (analytic grade, Promega™) with a 100 pb DNA marker (Promega™), using ethidium bromide staining (0.5 μg/ml) and a data image Analyzer with UV trans-illuminator (Gel Doc option, BioRad™).

### Data analysis

All quantitative data were encoded in a data sheet and processed using SPSS 12.0 for Windows and XLSTAT 2007. A 2 × 2 table was created to analyze association values between the three diagnosis methods. The following indicators for Parascreen™ RDT detection were calculated, using either PCR or expert microscopy (EM) as the gold standard: (95% CI): sensitivity (Se), specificity (Sp), positive (PV+) and negative predictive values (PV-), positive (LR+) and negative likehood ratio (LR-). All non-*P falciparum *infections were confirmed to be *P. vivax *by microscopy and PCR (no detection of *P. malariae *and *P. ovale*).

### Ethical considerations

This study was approved by the Ethics Review Board of the Universidad Peruana Cayetano Heredia, Lima, Peru (Code SIDISI: 051675). Before any sample was taken, the participants signed an informed consent.

## Results

A total of 332 symptomatic malaria patients were included in this study. The mean age was 32 ± 16 years. The results of EM, PCR and Parascreen™ are shown in Table 1. With EM, there were 234 negative and 98 positive slides, 83 *P. vivax *infections with parasite density between 24 to 17,385 parasites/μl (2,662,323 ± 3,083.7) and 15 *P. falciparum *infections with parasite density between 36 to 36,457 parasites/μl (4,622.8 ± 8,920). With PCR, there were 232 negative and 100 positive samples, 88 *P. vivax*, 11 *P. falciparum *and one mixed infection (*P. falciparum *and *P. vivax*). With Parascreen™, there were 250 negative and 82 positive samples, 70 *P. vivax *infections and 12 *P. falciparum*.

**Table 1:** Parascreen™ diagnosis results and comparison with diagnosis by PCR and EM.

**Results by Parascreen™**		**PCR**				**EM**		
			
		**Negative**	***P.vivax***	***P.falciparum***	**Mixed**	**Negative**	***P.vivax***	***P.falciparum***
		
Negative	**250**	230	19	1	0	230	16	4
non-*P. falciparum*	**70**	1	67	1	1	3	64	3
*P. falciparum*	**12**	1	2	9	0	1	3	8
								
Total	**332**	**232**	**88**	**11**	1	**234**	**83**	**15**

EM was first compared to PCR as gold standard, showing the following indicators: for detection of *P. falciparum *malaria, Se: 90.9% and Sp: 96.8%; and for non-*P. falciparum *malaria, Se: 90.9% and Sp: 98.76%. A discrepancy in species diagnosis between PCR and EM diagnosis was observed in five cases (including one mixed infection detected by PCR). Furthermore, four cases with negative diagnosis by EM were detected positive by PCR, and two cases with negative results by PCR tested positive by EM.

Table 2 presents the calculated indicators when Parascreen™ was compared with EM and PCR. When EM was used as gold standard, Parascreen™ had, for *P. falciparum *detection the following indicators: Se: 53.5%, Sp: 98.7%, PV+: 66.7%, PV-: 97.8%, LR+: 42.27 and LR-:0.47; and for non-*P. falciparum *malaria Se: 77.1%, Sp: 97.6%, PV+: 91.4%, PV-: 92.7%, LR+: 32.0 and LR-: 0.22. *P. falciparum *diagnosis using Parascreen™ had a high proportion of false negatives 46.7% [95% CI (24.8-69.9)].

**Table 2:** Comparative indicators of Parascreen™, when using PCR and EM as gold standards.

gold standard	Results by Parascreen™						
	*Plasmodium* Species	Sensitivity [%(95%CI)]^a^	Specificity [%(95%CI)]	PPV^b^ [%(95%CI)]	PPN^c^ [%(95%CI)]	Likehood ratio	
						Positive test	Negative test
**EM**	non-*P. falciparum*	77.1 (67.0-84.4)	97.6 (94.8-98.9)	91.4 (82.5-96.0)	92.7 (89.0-95.3)	32	0.22
	*P. falciparum*	53.5 (30.1-75.2)	98.7 (96.8-99.5)	66.7 (39.1-86.2)	97.8 (95.6-98.9)	42.27	0.47

**PCR**	non-*P. falciparum*	76.1 (66.3-83.8)	99.2 (97.0-99.8)	97.1 (90.0-99.2)	92 (88.1-94.7)	92.51	0.24
	*P. falciparum*	81.8 (52.3-94.9)	99.1 (97.3-99.7)	75 (46.8-91.1)	99.4 (97.7-99.8)	87.27	0.18

a.- Confidence interval at 95%.

b.- Positive predictive value.

c.- Negative predictive value.

When PCR was used as gold standard, Parascreen™ showed a better sensitivity for detection of *P. falciparum *and *P. vivax *infections. For *P. falciparum*, the indicators were: Se: 81.8%, Sp: 99.1%, PV+: 75%, PV-: 99.4, LR+: 87.27 and LR-: 0.18. For non-*P. falciparum *the following indicators were observed: Se: 76.1%, Sp: 99.2%, PV+: 97.1%, PV-: 92.0%, LR+: 92.51 and LR-: 0.24.

Parascreen™ had a low sensitivity (12.5%) for detection of *P. vivax *infections at parasite densities below 99 parasites/μl, which increased for the detection of parasite densities between 99 and 500 parasites/μl (sensitivity of 60%). For *P. falciparum *infections, parasite densities below 1,000 parasites/μl were detected with less sensitivity (25%) than parasite densities > 1,000 parasites/μl (sensitivity between 66.7% and 75.0%) (Table 3).

**Table 3:** Parascreen™ sensitivity, PPV and False-negative proportion at different parasitaemia determined by expert microscopy.

Parascreen™	Parasite density (parasites/ul)	N (Number of samples)	Sensitivity [%(95%CI)]	PPV [%(95%CI)]	False - negative proportion [%(95%CI)]
*P. vivax*					
	1-99	8	12.5 (2.2-47.1)	100	87.5 (52.9-97.8)
	100-499	15	60.0 (35.7-80.2)	100	40.0 (19.8-64.3)
	500-999	13	84.6 (57.8-95.7)	100	15.4 (4.3-42.2)
	1,000-4,999	35	91.4 (77.6-97.0)	100	8.6 (3.0-22.4)
	+ 5,000	12	91.7 (64.6-98.5)	100	8.3 (1.5-35.4)
*P. falciparum*					
	1-999	5	25.0 (4.6-69.9)	100	75.0 (30.1-95.4)
	1,000-1,999	3	66.7 (20.8-93.9)	100	33.3 (6.1-79.2)
	2,000-4,999	4	75.0 (30.1-95.4)	100	25.0 (4.6-69.9)
	+ 5,000	3	66.7 (20.8-93.9)	100	33.3 (6.1-79.2)

a,b.- Abbreviations as in Table 2

## Discussion

This study, conducted in Iquitos, Department of Loreto, showed an unacceptable performance of Parascreen™ for malaria diagnosis under the field conditions found in the Peruvian Amazon. Even though the RDT was of simple training and use for the local health staff, it showed variable indicators when compared with EM and PCR, depending on the *Plasmodium *species (*P. vivax *or *P. falciparum*) and parasitaemia of the infections.

In this study, Parascreen™ sensitivity for malaria infection was lower than the 100% sensitivity obtained in a validity assessment done by Zephyr Biomedical System [23]. In studies performed in Kenya [19] and India [21] the sensitivity values were 94.0% and 96.3% respectively. A study carried out in Ethiopia [20] describes similar results to the ones obtained in this study. In comparison with other RDTs, Parascreen™ sensitivity for *P. falciparum *infection was lower than that obtained with OptiMAL™ which detects *Plasmodium *lactate deshydrogenase. HRP2-based RDTs such as Parascreen™ have also been evaluated in various studies in South American countries [25-28]. The sensitivity and specificity for *P. falciparum *and *P. vivax *infections observed in this study were better than those reported with AMRAD fast test ICT *P.f/P v *in Peru [6], and similar to those obtained with Binax NOW™ fast test ICT *P.f/P.v *in Colombia, but lower than those observed with NOW-Malaria-ICT RDT in an evaluation study in Colombia [18] and in various other studies of OptiMAL IT [6,10,11,13,26].

The likelihood ratio (Lr) is independent of the prevalence of the disease in the population, and can be used to determine the accuracy of positive or negative test results [29]. In this study, high likelihood ratios of a positive test (Lr+), above 10, were found for *P. falciparum *and non-*P. falciparum *infections; confirming that patients with positive tests would have a high probability to be infected.

On the contrary, the observed likelihood ratios of a negative Parascreen™ test (Lr-), with values above 0.1, indicated a high risk of error when excluding malaria diagnosis in patients with negative tests. Similar findings were found in an evaluation study of OptiMAL™ in febrile travelers returning from malaria endemic countries, especially with *P. falciparum *infections [30].

More generally, it should be noted that PCR could be more relevant as a reference method for the evaluation of non-microscope procedures like RDTs [8,14,31], but the indicators still need to be considered with reference to EM, for inter-study comparisons.

RDTs generally achieve a sensitivity of > 90% at densities above 100 parasites per μL of blood, and the sensitivity decreases markedly below that level of parasite density.

The low number of *P. falciparum *infections in this study could explain the low Parascreen™ sensitivity for *P. falciparum *infections, as well as the sensitivity discrepancies when using EM and/or PCR as gold standards. Other possible factors, could consist in the genetic diversity of the parasite antigen HRP-2 [32], the existence of parasites that do not carry the *pfhrp2 *gene in this region [17,33] and the lack of monoclonal antibodies specificity used by the RDT to detect the HRP- 2 antigen [34]. When this study was carried out in 2006, and this RDT chosen for validation, the existence of *P. falciparum *isolates lacking the *pfhrp2 *gene in this region was still unknown and unpredictable, but now this recent discovery has to be taken into account when interpreting results of RDT evaluation studies in general.

## Conclusions

The present study showed that Parascreen™ sensitivity was comparable to EM and PCR at parasitaemia levels ≥100 parasites/μl in *P. vivax *infections and ≥1,000 parasites/μl for *P. falciparum *infections. In general, Parascreen™ sensitivity was low for *Plasmodium *infections, especially for fatal infections like *P. falciparum*. This RDT was not acceptable for malaria diagnosis under the field conditions found in the Peruvian Amazon, even though it was easy to learn and to use by the local health staff. The choice of a RDT for Amazonian countries such as Peru, should consider the local malaria incidence and predominance of *Plasmodium *species, as well as the genetic characteristics of the parasites, especially the existence in this region of *P. falciparum *field isolates lacking the *pfhrp2 *gene.

## Competing interests

The authors declare that they have no competing interests.

## Authors' contributions

AR, TG, ALL-C, JE and HG participated in designing the study. TG directed the field study. JB performed laboratory work. TG, AR and JB verified data. AR and JB analyzed data and participated in the manuscript preparation. DG was involved at all stages of this study and led the manuscript preparation. All authors have read and approved the final manuscript.
